# Main bronchial sleeve resection with pulmonary conservation

**Published:** 2008-04-15

**Authors:** Cordos Ioan, Bolca Ciprian, Paleru Cristian

**Affiliations:** * Surgery Toracic Clinic – Central Military Emergency Universitary Hospital“Dr. Carol Davila”

**Keywords:** main bronchial sleeve resection, lung preservation, bronchial stenosis, bronchial carcinoid, metachronous squamous cell carcinoma, bronchial fracture

## Abstract

Cerfolio et al defines main bronchial sleeve resection as circumferential resection of either right or left main bronchus and presents the indications for main bronchial sleeve resection – localized tumor lesions, benign stenosis, bronchial localized lithiasis, posttraumatic stenosis – stating that in carefully selected patients, the procedure is safe and efficient.

This article aims to present the experience of our clinic regarding 4 cases of primitive bronchial disease for which a main bronchial sleeve resection with pulmonary conservation were performed between 2005 and 2006.

The reduced number of cases allows us express the opinion that in well evaluated cases – where bronchoscopic exam is essential – the main bronchial sleeve resection and full lung preservation is a procedure that can be made with excellent results for the patient. Life quality is superior to that of cases with pneumonectomy which can be a disease by itself. An adequate technique, the respect for tissues, radical oncologic approach, absorbable monofilament suture material, as well as the protection of the anastomosis with viable tissue from vicinity (pleura, pericardial fat, intercostal muscles) are followed by the best results.

## Introduction

Cerfolio et al defines main bronchial sleeve resection as circumferential resection of either right or left main bronchus. In 1947 Sir Clement Price Thomas was the first one to perform this type of surgical procedure and all the other authors recognize this. When the disease incriminates only the main bronchus (especially in benign lesions or in cases when the respiratory function is compromised and another oncologic surgical procedure would jeopardize patient life) the resection of main bronchus is indicated.

## Patients and methods

Between June 2005 and June 2006 we performed 4 surgical procedures for primitive bronchus diseases of different aetiology: one patient with COPD presented a metachronous squamous cell carcinoma, with functional respiratory tests that didn’t allow a completion right pneumonectomy; one patient with a typical carcinoid with origin in the distal part of right primitive bronchus; a patient with posttraumatic complete stenosis of the left main bronchus and one patient with a ganglio-bronchial fistula that produced an incomplete stenosis of the left main bronchus.

## Case 1

**Fig. 1:**
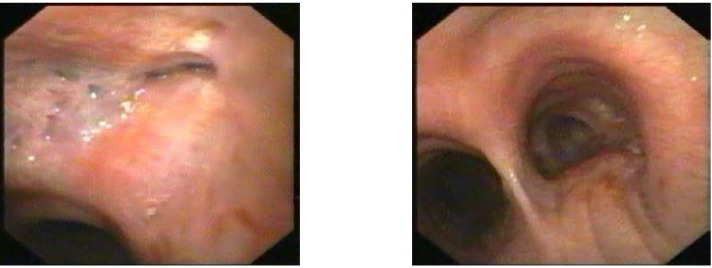


**Fig. 2:**
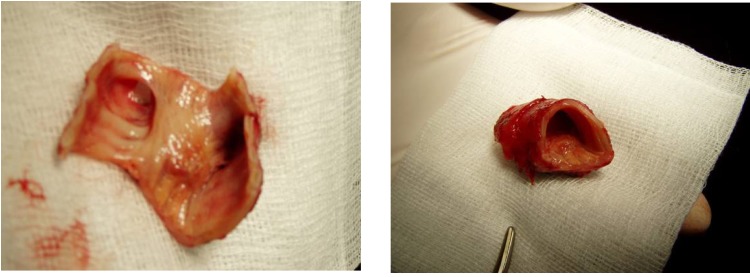


**Fig. 3:**
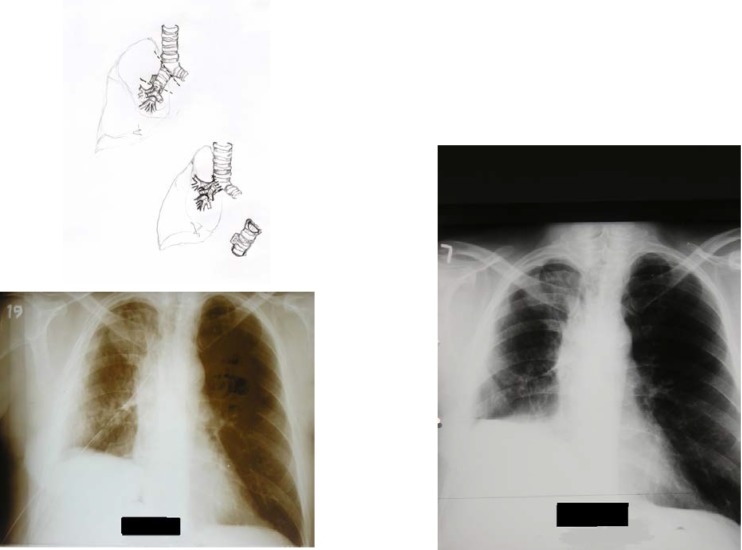


**Fig. 4:**
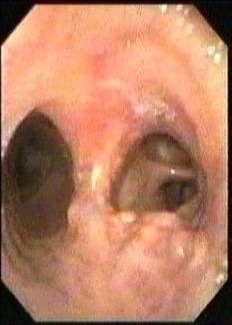


A 53 year old male, operated three years ago (right upper lobectomy) for a squamous cell carcinoma with medium differentiation stage I B (T2NOMO), presented for irritative cough, asthenia and small quantity haemoptysis. No evolutive lesions were detected by radiological examination and computer tomography of the chest. An infiltrative lesion of 4/5 mm is revealed by bronchoscopic examination (**Fig. 1**). We performed a biopsy and a histopathological examination and the result was well differentiated squamous cell carcinoma. Secondary diagnoses of the patient were chronic hepatitis and COPD. The therapeutic options were: chemotherapy or completion pneumonectomy, but the last one was not indicated because of the low functional respiratory status. We decided to perform a main and intermediary sleeve bronchial resection with anastomosis between the distal part of the intermediary bronchus and trachea, with a good outcome (**Fig. 2**, **3**). One year later, the clinical, radiological and bronchoscopic examinations were normal (**Fig. 4**)

## Case 2

**Fig. 5:**
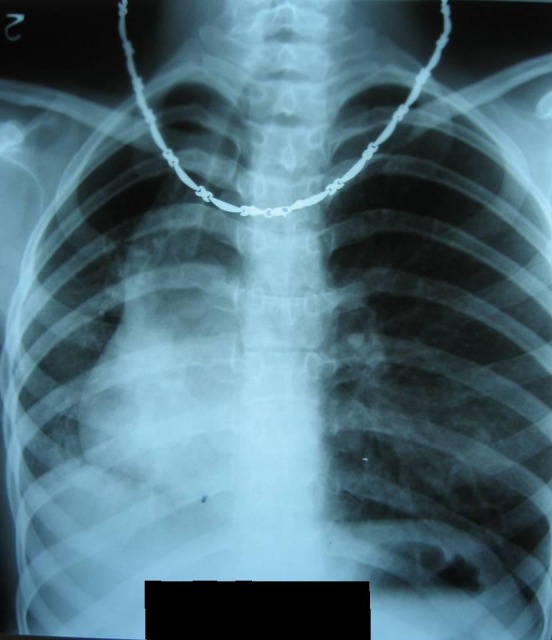


**Fig. 6:**
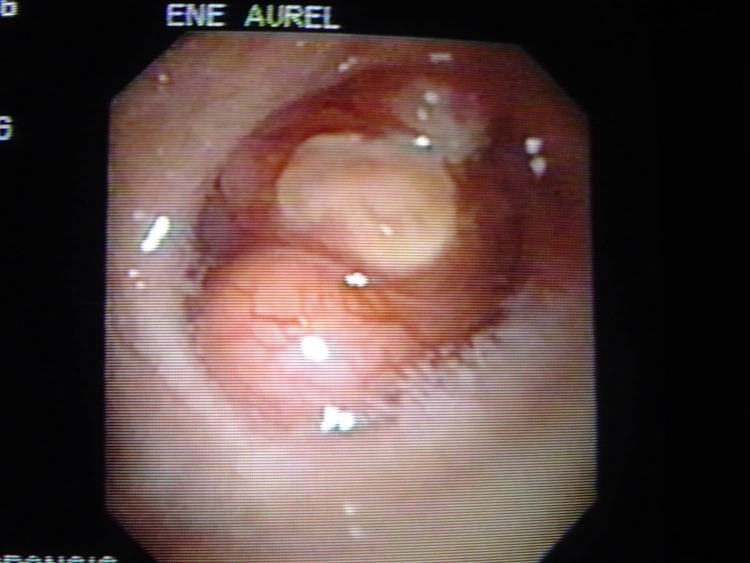


**Fig. 7:**
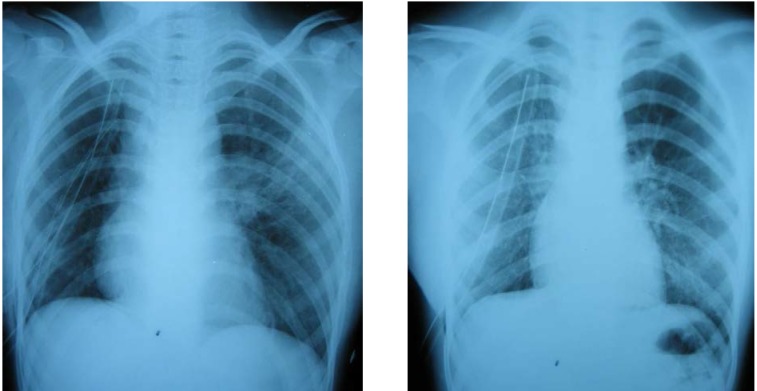


A 22 year old female was admitted in hospital for pain in the right hemithorax, dyspnoea and small quantity haemoptysis, which lasted for more than a month. Progressive dyspnoea made the patient rush to the hospital. Standard radiological examination revealed mediastinal deviation to the right, initially diagnosed as dextrocardia (**Fig. 5**). A smooth tumoral process, with rich vascularisation that obstructed almost all of the right main bronchus was revealed by bronchoscopic examination (**Fig. 6**). The tumor extended upward into the trachea. The diagnosis was bronchial carcinoid. The surgical procedure performed was main right bronchial sleeve resection with bronchotracheal anastomosis (**Fig. 7**). Histopathological examination of the tumor and hilum adenopathies indicated the presence of typical carcinoid without invasion in regional lymph nodes. After 6 days with favorable postoperative evolution, the patient left the hospital. Bronchoscopic examination after 3 months revealed healing without stenosis.

## Case 3

**Fig. 8:**
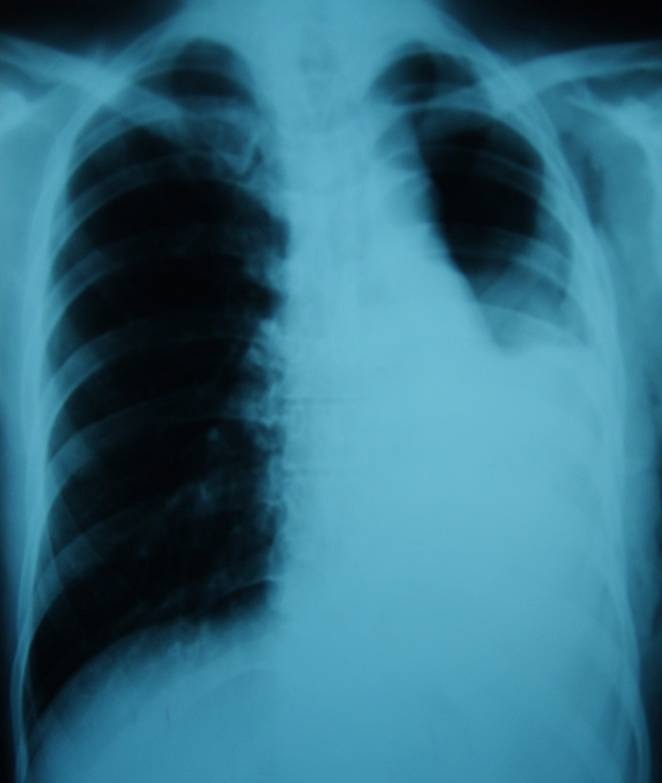


**Fig. 9:**
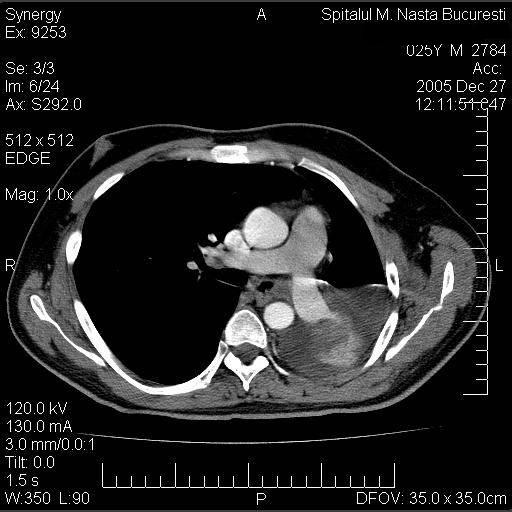


**Fig. 10:**
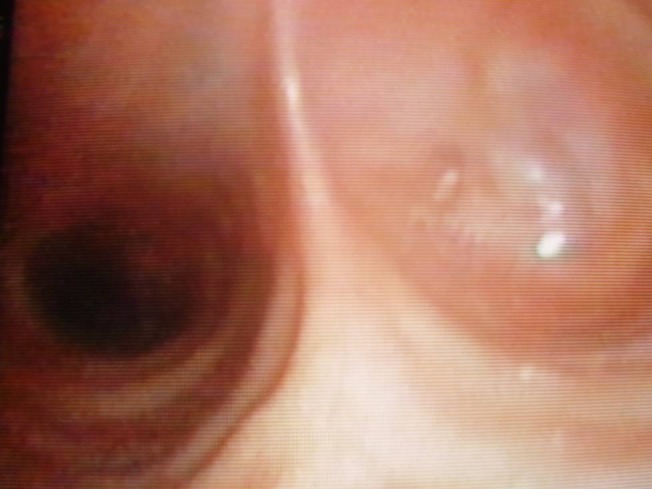


**Fig. 11:**
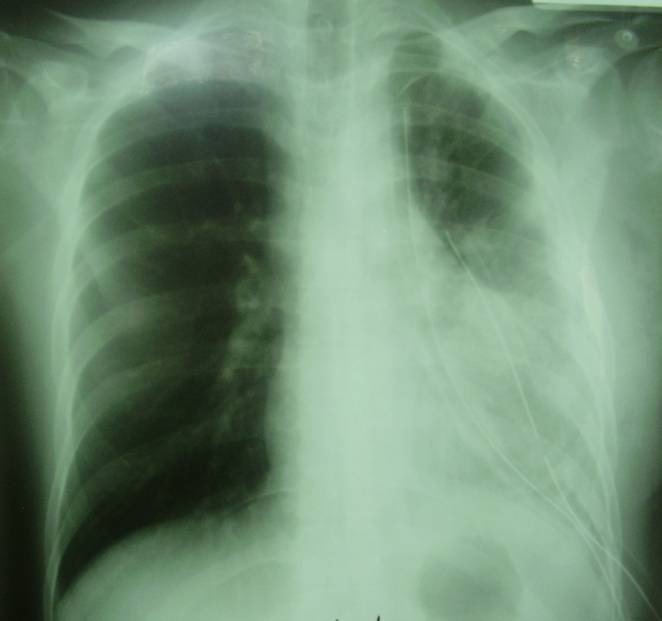


**Fig. 12:**
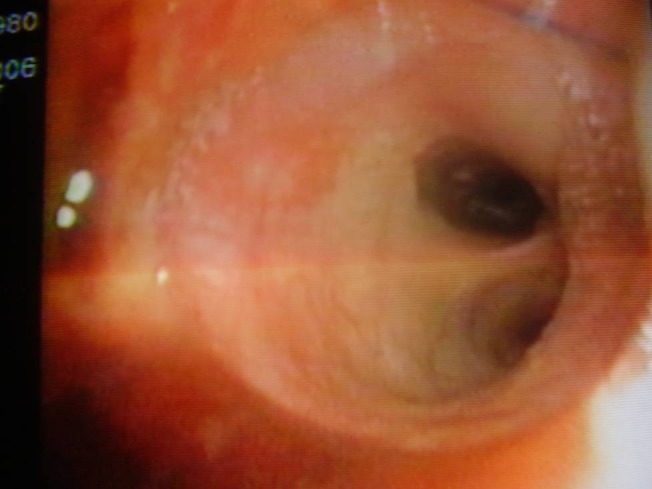


A 25 year old male, with polytraumatic lesions, in coma after car crash, is intubated and mechanically ventilated for 4 days in other hospital unit. The lesions found were: acute closed cranio-cerebral trauma, thoracic trauma with multiple rib fractures and left haemopneumothorax, right femur fracture. Even if a chest tube was placed (in the left hemithorax) that evacuated about 1 liter of incoagulable blood, the lung would not expand. 30 days after trauma, the patient was conscious and still presented pain in the left hemithorax, radiological evident hydropneumothorax (**Fig. 8**) and medium effort dyspnoea. The CT scan suspected a left main bronchus fracture and also revealed a total left pulmonary atelectasis (**Fig. 9**). The patient was brought by transfer to our clinic. Bronchoscopic exam showed a complete left main bronchial stenosis beginning with the third cartilage (**Fig. 10**). The normal clinical status of the patient allowed us to perform a main bronchial sleeve resection and pulmonary decortication. The lung that expanded normally at the end of the procedure was preserved. We mentioned that atelectasis lasted for 3 months before January 2006, the date of surgical procedure. The postoperative evolution was favorable and the bronchoscopic exam indicated a normal permeability and diameter of the anastomosis (**Fig. 11**, **12**).

## Case 4

**Fig. 13:**
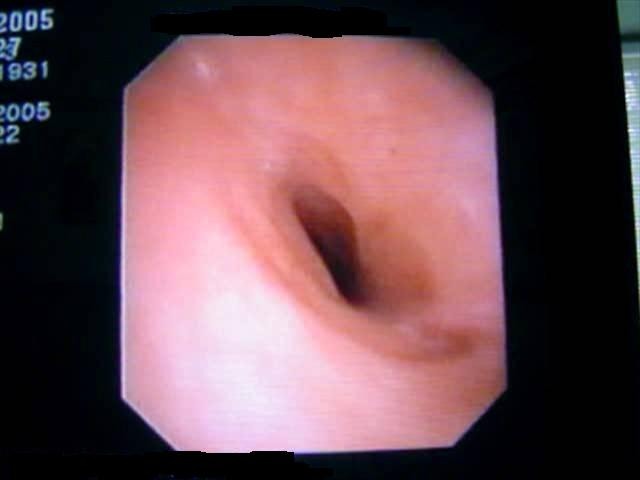


**Fig. 14:**
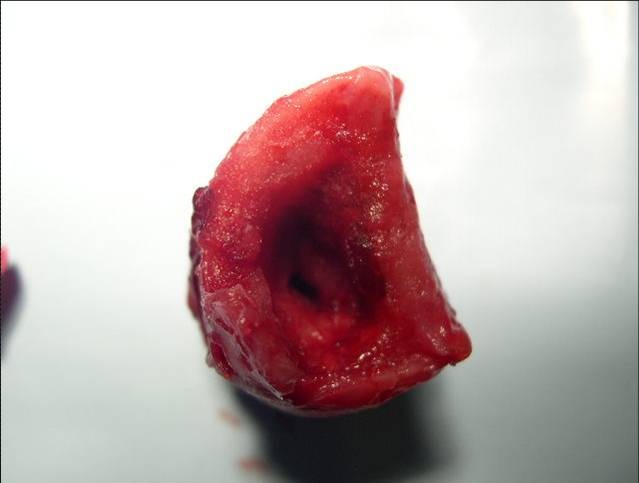


**Fig. 15:**
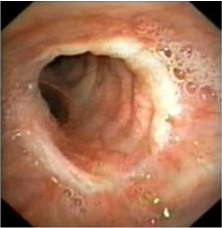


A 32 year old male in evidence with a diagnosis of tuberculosis 2 years prior was admitted to the pneumology clinic with repetitive left basal pneumonic processes. The most important symptoms that made the patient rush to the hospital were: irritative cough with medium quantity of muco-purulent expectoration, subfebrility and small haemoptysis. Standard thoracic radiological exam showed an enlarged left pulmonary hilum and sequelas of fibro-nodular lesions in the upper lobe of the same side. The bronchoscopic exam revealed a 5mm stenosis in diameter beginning with the 4th ring of the left main bronchus that did not allow the passage of the bronchoscope (**Fig. 13**). The surrounding bronchial mucosa was congestive and a purulent secretion was draining out of the stenotic lumen. Bacteriology exam certified the absence of Koch Bacillus. CT exam showed the quasi normal aspect of the left distal main bronchus and certified the presence of fibrous sequelas in both upper lobes. We proposed and performed a left main bronchial sleeve resection of about 2.5cm followed by a favorable postoperative evolution (**Fig. 14**). On the 7th day after surgery, the bronchoscopic exam revealed the integrity of the anastomosis (**Fig. 15**). The patient returned to the pneumology clinic to continue the tuberculostatic treatment.

In all four cases, the diagnosis was established by the bronchoscopic examination which allowed the description and biopsy of lesions. In 3 cases the CT examination correctly evaluated the status of pulmonary parenchyma as well as the status of local regional lymph nodes. The surgical approach was lateral thoracotomy in the 5th intercostal space, an approach that offers good and sufficient light for the safe dissection of the hilum. In the first case, the azygos vein was divided due to the adherences between the vein and the tracheobronchial angle following the previous procedure. On the left side, the arterial ligament (ductum arteriosum Botallo) was divided to enlarge the space under the aortic arch for the resection and the anastomosis. The anastomosis was performed using absorbable 3.0 threads (polidioxanone) for the anterior and posterior aspects of the anastomosis. The suture was covered with mediastinal pleura in the 1st and 2nd case (on the right side) and with pediculated pericardial fat for the 4th case (on the left side). The main bronchial sleeve resections were between 1cm (for typical carcinoid) and 3cm (for metachronous squamous cell carcinoma). A radiologic examination was performed daily and a bronchoscopic exam in the 7th and the 30th day after the procedure. 

## Results

The postoperative outcomes were favorable in all 4 cases with a minimum of 6 days of hospital care (case 2) and a maximum of 9 days of hospital care (case 4). Six months after procedures, the radiologic and bronchologic examination had no pathological modifications. The 1 year follow-up CT and bronchoscopy in the case of the patient with metachronous squamous cell carcinoma certified the absence of the disease.

## Discussions

Cerfolio, et al. presents the indications for main bronchial sleeve resection – localized tumor lesions, benign stenosis, bronchial localized lithiasis, posttraumatic stenosis – stating that in carefully selected patients, the procedure is safe and efficient. In our cases, the clinical diagnosis was suggested by irritative cough and small haemoptysis that demanded further investigations. The bronchoscopy examination played a major role in establishing the topography of the lesions, the histopathology diagnosis, evaluation of surrounding tissue status and last but not least the status of future anastomosis components. The bronchoscopy’s role is preoperative and also postoperative by cleaning the bronchus distal from the anastomosis. In time, bronchoscopy predicts the risk of restenosis and is a method of treatment by dilatation and stenting as well. 

In Newton’s et. al. study on 27 main bronchial sleeve resections, 15 for cancer and 12 for different benign lesions, even if no perioperative death was observed, in 2 of the benign lesion cases there was a need for serial dilatations and in one case the patient was successfully reresected. Further postoperative complications described by these authors were: prolonged air loss, transient paresis of the recurrent nerve, atelectasis and pneumonia.

Taking into consideration the diagnostic criteria for metachronous cancer recommended by Joe B. Putnam jr. – the different histological type, a 24 months free from disease interval and the presence of neoplasia in another lobe or contralateral lung – we classified case 1 in the category of metachronous cancer.

In our case we considered that the limited resection of the bronchial axis was sufficient at a patient that had associated pathology – chronic hepatitis and COPD – especially when CT scan didn’t show any extra bronchial extension of the neoplasm (in immediate vicinity or at distance).

The methods of treatment in carcinoid tumors are disputed between endoscopists and surgeons. Ducrocq et al. states that the bronchoplastic methods are a valid option for earlier stages. Typical carcinoids with a small base of implant – pediculated – can be treated by interventional bronchoscopy (Sutedja et al. 1995). We chose a surgical procedure in case 2 for several reasons: the presence of a large tumor without a clear endoscopic image of the implant base, the acute respiratory failure which imposed an emergency intervention, the fear for massive haemorrhage during endoscopic resection maneuvers, the impossibility to evaluate the lymph node invasion by means other than surgical biopsy. Histology examination proved a typical carcinoid and no lymph node metastasis. Postoperatively, the dyspnoea remitted completely. Despite the 3 months atelectasis, the left pulmonary parenchyma was conserved in such a good condition that after the left main bronchial sleeve resection, the lung expanded completely in good condition almost immediately. 

The difficulty of reshaping the airway in the narrow aortic-pulmonary window was diminished by sectioning of the Botallo arterial ligament between ligatures. This maneuver allowed antero-inferior retraction of the left pulmonary artery and visualization of the tracheal bifurcation. In the 4th case this step was particularly difficult with a lot of blood loss due to the dissection that was worsened by inflammatory lymph nodes from the aortic-pulmonary window. Consequently, the anastomosis seemed to be practiced in a low quality tissue. In such conditions we found it opportune to cover the anastomosis with pediculated pericardial fat as in all left pneumectomies. This maneuver offered protection against bronchial anastomosis leaks even if some recent works (medicine based evidence) did not support this affirmation.

The reduced number of cases does not allow us to conclude, but only to express the opinion that in well evaluated cases – where the bronchoscopic exam is essential – the **main bronchial sleeve resection** and full lung preservation is a procedure that can be made with excellent results for the patient. Life quality is superior to that of cases with pneumonectomy which can be a disease by itself. An adequate technique, the respect for tissues, radical oncologic approach, absorbable monofilament suture material, as well as the protection of the anastomosis with viable tissue from the vicinity (pleura, pericardial fat, intercostal muscles) are followed by the best results.
